# New Insights on Chromosome Diversification in Malagasy Chameleons

**DOI:** 10.3390/ani14192818

**Published:** 2024-09-30

**Authors:** Marcello Mezzasalma, Gaetano Odierna, Rachele Macirella, Elvira Brunelli

**Affiliations:** 1 Department of Biology, Ecology and Earth Science, University of Calabria, Via P. Bucci 4/B, 87036 Rende, Italy; rachele.macirella@unical.it (R.M.); elvira.brunelli@unical.it (E.B.); 2 Independent Researcher, Via Michelangelo 123, 81031 Aversa, Italy; gaetanodierna@gmail.com

**Keywords:** cytogenetics, evolution, karyotype, Madagascar, reptiles

## Abstract

**Simple Summary:**

Chromosome diversification represents a fundamental driver of biological evolution. Here we present and discuss the results of a comparative chromosome analysis on different species of the Malagasy chameleons of the genera *Brookesia* and *Furcifer*. We show that the study species are characterized by different karyotypes in terms of chromosome number (2n = 36–22), ratio of micro- and macrochromosomes, and the variable presence of differentiated sex chromosomes. Considering our new data together with those from previous studies, we describe a chromosome evolutionary scenario in the studied taxa. Specifically, while genus *Brookesia* has a fixed chromosome number (2n = 36) and no differentiated sex chromosomes, which corresponds to the hypothesized ancestral chameleon karyotype, *Furcifer* shows a high diversity in chromosome number (2n = 20–34), morphology, and the independent raising of simple and multiple sex chromosomes with female heterogamety. The karyotypes in *Furcifer* with a relatively low chromosome count likely evolved from a chromosome complement similar to that of *F. balteatus* (2n = 34), mostly via a progressive number of chromosome fusions involving distinct micro- and macrochromosome pairs. Similarly, the diversification of simple and multiple sex chromosome systems occurred in *Furcifer* via independent (non-homologous) sex chromosome-autosome fusions and heterochromatinization.

**Abstract:**

In this work, we performed a preliminary molecular analysis and a comparative cytogenetic study on 5 different species of Malagasy chameleons of the genus *Brookesia* (*B. superciliaris*) and *Furcifer* (*F. balteautus*, *F. petteri*, *F. major* and *F. minor*). A DNA barcoding analysis was first carried out on the study samples using a fragment of the mitochondrial gene coding for the cytochrome oxidase subunit 1 (COI) in order to assess the taxonomic identity of the available biological material. Subsequently, we performed on the studied individuals a chromosome analysis with standard karyotyping (5% Giemsa solution at pH 7) and sequential C-banding + Giemsa, + CMA_3_, and + DAPI. The results obtained indicate that the studied species are characterized by a different chromosome number and a variable heterochromatin content and distribution, with or without differentiated sex chromosomes. In particular, *B. superciliaris* (2n = 36) and *F. balteatus* (2n = 34) showed a similar karyotype with 6 macro- and 12–11 microchromosome pairs, without differentiated sex chromosomes. In turn, *F. petteri*, *F. major*, and *F. minor* showed a karyotype with a reduced chromosome number (2n = 22–24) and a differentiated sex chromosome system with female heterogamety (ZZ/ZW). Adding our newly generated data to those available from the literature, we highlight that the remarkable chromosomal diversification of the genus *Furcifer* was likely driven by non-homologous chromosome fusions, including autosome–autosome, Z–autosome, and W–autosome fusions. The results of this process resulted in a progressive reduction in the chromosome number and partially homologous sex chromosomes of different shapes and sizes.

## 1. Introduction

The diversification of karyotypes via macromutations (or chromosome mutations) affects biological evolution by promoting population divergence and speciation and through the diversification of genetic sex determination systems (see, e.g., [1,2]).

Squamate reptiles, with more than 11,500 described species, represent one of the most taxonomically diversified groups of vertebrates [3]. Furthermore, their diversity at the species level and at higher taxonomic ranks is reflected by a karyotype variability that is unparalleled in other tetrapods. Specifically, karyotype diversity in squamate reptiles encompasses chromosome number (including phylogenetically related diploids with variable chromosome count as well as hybridogenetic triploids), morphology, a highly variable localization of chromosome markers, and the independent insurgence of simple or multiple sex chromosome systems (genetic sex determination) with female or male heterogamety at various levels of diversification (from mostly pseudoautosomal to highly heteromorphic and/or heterochromatic sex chromosomes (see, e.g., [4,5,6]). The occurrence of such high diversity, along with the possibility to identify potential plesiomorphic and apomorphic chromosome states, provides the opportunity to better understand different trends and mechanisms of karyotype and more in general of biological evolution (see, e.g., [6,7,8]).

Among squamates, several previous experimental analyses highlighted that the family Chamaeleonidae represents a particularly interesting taxonomic group in evolutionary cytogenetics (see e.g., [8,9,10,11,12]). In fact, multiple chromosome evolutionary trends have been identified among chameleons of different genera, including recurrent independent reductions in the chromosome number and the progressive reduction in the microchromosome number. These dynamics are exceptionally evident in the genus *Furcifer*, which also alternatively exhibits in different clades simple and multiple sex chromosome systems with female heterogamety [9,10,11,12].

Madagascar is a unique subcontinental landmass in terms of flora, fauna, geological, and environmental characteristics and has been historically considered a natural laboratory for the studies on biodiversity and evolutionary dynamics (see, e.g., [13,14,15]). The extraordinary species richness of the different Malagasy ecoregions includes six families of lizards and six families of snakes with more than 450 species and several undescribed lineages [3,16]. Among them, the family Chamaeleonidae occurs in Madagascar with 4 genera (*Brookesia*, *Palleon*, *Calumma,* and *Furcifer*) and more than 55 described species that are endemic to the island and neighboring archipelagos [3].

In this study, we performed a preliminary DNA barcoding and a comparative cytogenetic analysis with standard karyotyping and chromosome banding methods on four *Furcifer* (*F. balteatus*, *F. petteri*, *F. major* and *F. minor*) and one *Brookesia* species (*B. superciliaris*). These species were chosen considering their different chromosome complement and the possible occurrence of hypothesized ancestral and derivate autosomal and sex chromosome states (see [12]), which would help to better understand karyotype evolutionary dynamics. The DNA barcoding analysis was performed considering the difficult taxonomic attribution of several Malagasy chameleon species, the possible occurrence of undescribed lineages (see also [12]), and to associate DNA sequences to the newly generated chromosome data.

Considering our new data along with previous experimental evidence, we highlight that either autosome and sex chromosome diversity in the genus *Furcifer* likely started from a karyotype similar to the hypothesized ancestral chameleon karyotype (2n = 36), which is fixed in *Brookesia* [12]. Finally, we describe a chromosome evolutionary scenario concerning the processes at the origin of the observed karyotype diversity in *Furcifer*, with a particular focus on the diversification of Z and W chromosomes in different evolutionary lineages.

## 2. Materials and Methods

### 2.1. Sampling

We examined a total of six individuals of 5 different taxa of Malagasy chameleons of the genera *Brookesia* (*B. superciliaris*) and *Furcifer* (*F. balteatus*, *F. petteri*, *F. major*, and *F. minor*).

A complete list of field numbers, sampling localities, and sex of all the studied samples is provided in Table 1, along with the taxonomic attribution of the studied taxa following the DNA barcoding analysis (see below). No individual was collected during the realization of this work, and all the studied biological material was sampled during fieldwork performed between 2002 and 2004 by various colleagues and used for other research purposes. After sampling, the individuals were treated with a 0.5 mg/mL colchicine solution (0.1 mL/10 g of total body weight). Cell suspensions were then prepared from tissue samples incubated for 30 min in a hypotonic solution of KCl 0.075 M + sodium citrate 0.5% (1:1) and fixed and conserved in Carnoy’s buffer (methanol/acetic acid, 3:1). The biological material was temporarily preserved at 4 °C and then moved to the laboratory, where it was stored and processed as reported below.

### 2.2. Molecular Analysis

A DNA barcoding analysis was performed on all the examined samples using a fragment of the mitochondrial gene coding for the cytochrome c oxidase subunit I (COI). This molecular marker has been previously tested and proposed as the standard gene fragment for DNA barcoding of Malagasy reptiles [17]. Extraction of genomic DNA was performed from the available biological material following the traditional phenol–chloroform method as reported by Sambrook et al. [18]. After DNA extraction, a fragment of the COI (max length of 664 bp) was amplified by PCR in a reaction volume of 25 µL using the primer pair RepCOI-F: 5′-TNTTMTCAACNAACCACAAAGA-3′ (forward) and RepCOI-R: 5′-ACTTCTGGRTGKCCAAARAATCA-3′ (reverse) [17]. PCR was carried out using the following parameters: initial denaturation at 94 °C for 3 min; 40 cycles of 94 °C for 40 s, 48.5 °C for 30 s, and 72 °C for 60 s; 72 °C for 7 min [17]. All the obtained amplicons were then sequenced in both directions using the BigDye Terminator v3.1 kit (ABI) and an automated capillary sequencer, ABI 377 (Applied Biosystems, Foster City, CA, USA). The resulting chromatograms were manually checked and edited using Chromas Lite 2.6.6 and BioEdit 7.7.1 [19]. All the newly generated DNA sequences were deposited in GenBank (accession numbers: PQ272538-43). To compare the newly obtained sequences with those already available from the literature and assess the taxonomic attribution of the samples studied, all the newly generated DNA sequences were blasted on GenBank. For taxonomic attribution, we considered the mean genetic distances reported by Nagy et al. [17] for Malagasy chameleons belonging to traditionally recognized “good” species (19.9% p-distance) and genetic sister clades (9.1% p-distance).

### 2.3. Cytogenetic Analysis

Chromosome metaphase plates were obtained from stored tissue samples and cell suspensions (see above) using the conventional air-drying method as described in Mezzasalma et al. [20].

Chromosomes were then processed according to standard karyotyping protocol (5% Giemsa solution at pH 7 for 10 min) and sequential C-banding + Giemsa and C-banding + fluorochromes. C-banding + Giemsa was realized according to Sumner [21] and C-banding + 4′,6-diamidino-2-phenylindole (DAPI) and Chromomycin A_3_ (CMA_3_), following Mezzasalma et al. [22]. Karyotype reconstruction was performed using an optical and an epifluorescence microscope (Axioscope Zeiss, Oberkochen, Germany) equipped with an image analysis system after scoring and recording at least ten metaphase plates per sample. Chromosomes were categorized into metacentric (m), submetacentric (sm), subtelocentric (st), and acrocentric (t) following the classification described in Levan et al. [23].

## 3. Results

### 3.1. Molecular Analysis

The PCR amplification of the selected fragment of the COI was successfully performed on all the studied individuals.

In particular, the two studied samples of *B. superciliaris* (GA 199 and GA A3) showed the same haplotype and a maximum identity score of about 90.5% with two conspecific sequences available on GenBank (AN: EF222187 and JQ909296). The COI sequence of the sample GA 379 belonging to *F. balteatus* showed a maximum identity score of about 99.5% with the only conspecific homologous sequence available online (AN: JQ909368). Similarly, the sequence from the female sample of *F. minor* (GA 490) showed a maximum identity score of about 99.7% with the only available sequence of the same species (AN: MN757877). The sequence from the sample GA 456 belonging to *F. major* showed an identity score of about 97–98.5% with three available homologous sequences of the same species (AN: MZ285478, MH063342, and MH063343). However, the same sequence also showed an identity score of about 95.5% with a sequence ascribed to *F. viridis* (AN: JQ909372) and of about 92.8–93.3% with three sequences ascribed to *F. lateralis* (AN: MN757875, JQ909371, and MZ285476).

### 3.2. Cytogenetic Analysis

All the cytogenetic techniques were successfully performed on all the studied individuals. The only exceptions were represented by the male sample of *B. superciliaris* and the female sample of *F. major*, which were analyzed only with C-banding + fluorochromes.

**Figure 1:**
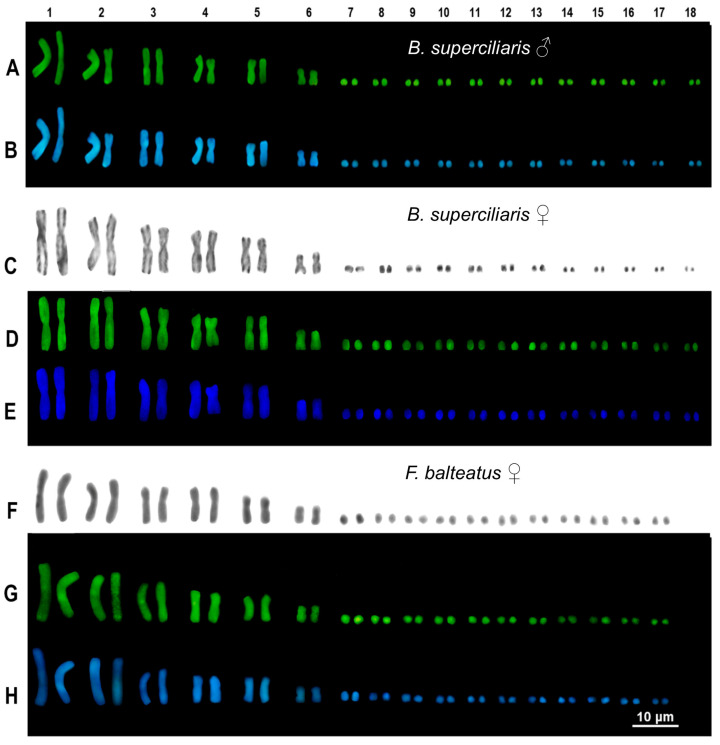
Karyotypes of the studied male (**A**,**B**) and female (**C**–**E**) of *B. superciliaris* and of the female individual of *F. balteatus* with C-banding + Giemsa (**C**,**F**), + CMA_3_ (**A**,**D**,**G**). and + DAPI (**B**,**E**,**H**). Scale bar applies to all images.

The two studied individuals of *B. superciliaris* (one male and one female) showed a karyotype composed of 2n = 36 chromosomes with six macrochromosome pairs and twelve microchromosome pairs (Figure 1).

All the macrochromosome pairs were metacentric, and no dimensional or morphological differences were found between sexes. After C-banding, *B. superciliaris* showed a limited amount of heterochromatin, mostly recognizable as telomeric, centromeric, and pericentromeric spots (Figure 1). No largely heterochromatic (heterogametic) sex chromosome was identified in either the male or the female individual analyzed in this study (Figure 1).

The female individual of *F. balteatus* showed a karyotype of 2n = 34 chromosomes with 6 macrochromosome pairs and eleven microchromosome pairs (Figure 1). Similarly, to *B. superciliaris*, all the macrochromosome pairs were biarmed (metacentric), and no dimensional or morphological differences were found among pairs. A limited content of heterochromatin was detected mostly as telomeric and centromeric blocks (Figure 1). No heteromorphic or largely heterochromatic chromosome was detected following C-banding + Giemsa or C-banding + fluorochromes (Figure 1).

**Figure 2:**
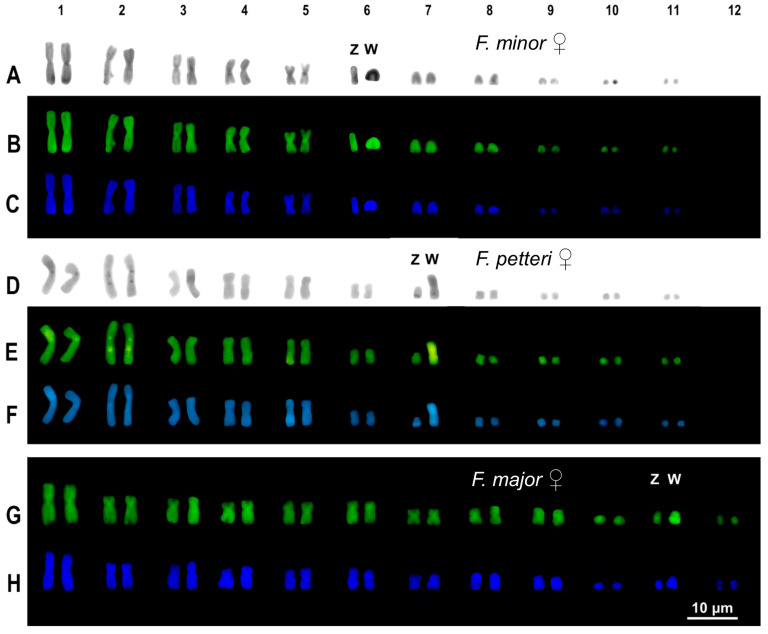
Karyotypes of the studied females of *F. minor*, (**A**–**C**), *F. petteri* (**D**–**F**), and *F. major* (**G**,**H**) with C-banding + Giemsa (**A**,**D**), + CMA_3_ (**B**,**E**,**G**), and + DAPI (**C**,**F**,**H**). Scale bar applies to all images.

The available female sample of *F. minor* showed a karyotype composed of 2n = 22 chromosomes with eight macro- and three microchromosome pairs (Figure 2).

Among macrochromosomes, the first five pairs were metacentric, while the 6th, 7th, and 8th pairs were composed of acrocentric elements (Figure 2). No dimensional heteromorphism was detected among any chromosome pair of *F. minor*. Sequential C-banding + Giemsa and + fluorochromes evidenced that one of the elements of the 6th pair was almost completely heterochromatic and was therefore identified as the W chromosome (Figure 2).

The karyotype of the female individual of *F. petteri* is composed of 2n = 22 chromosomes with eight macrochromosome pairs and three microchromosome pairs (Figure 2). Among macrochromosomes, 7 pairs were metacentric with the exception of the 5th pair, which was submetacentric (Figure 2). The 7th macrochromosome pair appeared heteromorphic, with one element much larger than the other (Figure 2). The analysis with sequential C-banding (C-banding + Giemsa and C-banding + fluorochromes) evidenced that the larger element is highly heterochromatic, probably corresponding to a W chromosome (Figure 2). Furthermore, additional heterochromatic spots were identified on centromeric, pericentromeric, and telomeric regions of several chromosome pairs, and they were generally more evident with C-banding + Giemsa and C-banding + CMA_3_ (Figure 2).

The studied female of *F. major* presented a karyotype of 2n = 24 chromosomes, with nine macrochromosome pairs and three microchromosome pairs (Figure 2). Seven macrochromosome pairs (1, 4, 5, 6, 7, 8, and 9) were metacentric, while pairs 2 and 3 were submetacentric (Figure 2). A dimensional heteromorphism was detected in a microchromosome pair, here tentatively identified as the 11th pair (Figure 2). Analysis with C-banding + CMA_3_ and + DAPI highlighted that the larger element of the 11th pair was largely heterochromatic and likely corresponds to the W sex chromosome (Figure 2).

**Figure 3:**
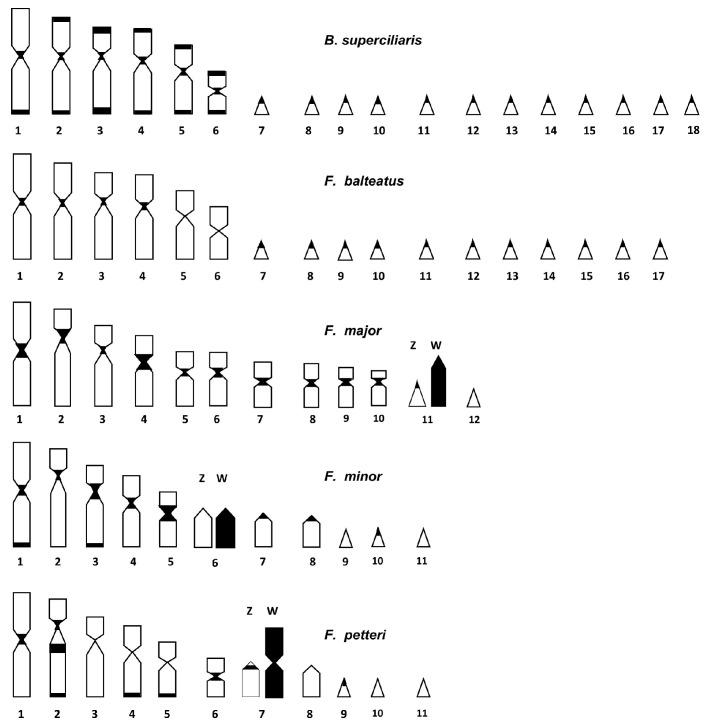
Karyograms of the studied species with heterochromatic regions highlighted in black and, when present, heteromorphic/heterochromatic sex chromosome pairs.

The comparison of our banding results revealed that heterochromatin is distributed in the study species mostly on centromeric and telomeric regions, and it is overall more evident in species with a lower chromosome number (e.g., *F. major*, *F. minor*, and *F. petteri*) (Figure 3).

## 4. Discussion

### 4.1. Molecular Taxonomic Attribution

Following the results obtained in the preliminary barcoding analysis, we are confident about the molecular taxonomic attribution of the studied samples provided in Table 1. In fact, all the individuals analyzed in the present study showed an uncorrected p-distance with homologous conspecific sequences that are close to or within the mean intraspecific distances found in the family Chamaeleonidae by Nagy et al. [17]. The only somewhat ambiguous result is represented by the molecular affinities of our and deposited COI sequences of *F. major* (see Results), also with samples reported as *F. viridis* and *F. lateralis* [11,17,24], which also fall within the above-mentioned mean intraspecific distances. It is possible that some closely related *Furcifer* species are characterized by a lower interspecific genetic distance than the mean values reported by Nagy et al. [17] or, alternatively, that some available sequences on GenBank have been erroneously identified. It should also be noted that the sampling locality of the *F. major* sample here studied is Belalanda (Table 1), which is not far away from Tanandava (the type locality of the species).

### 4.2. Cytogenetic Analysis

Recent cytogenetic studies highlighted that the family Chamaeleonidae is characterized by a remarkable karyological diversity in terms of total chromosome number, ratio of macro- and microchromosome pairs, chromosome morphology, and chromosome location of different cytogenetic markers (e.g., NORs, heterochromatic blocks, interstitial telomeric sequences) [9,10,11,12]. Furthermore, standard and molecular cytogenetic analysis coupled with phylogenetic comparative methods also indicated that the general chromosome evolution in chameleons proceeded toward an independent reduction of the total number of chromosomes in different evolutionary lineages, starting from an ancestral karyotype of 2n = 36 chromosomes with six macro- and twelve microchromosome pairs [12]. This mechanism mostly involved whole chromosome fusions (micro- to micro and micro- to macrochromosome translocations) [12].

Nevertheless, other evolutionary trajectories also occur in the family (e.g., chromosome conservatism or augment of the chromosome number), but are overall less represented in the chameleon phylogeny [12].

For example, in some genera such as *Brookesia* and *Palleon* (subfamily Brookesinae, which represents the sister groups to all other chameleons), as well as in *Kyniongia*, the ancestral karyotype of 2n = 36 chromosomes is fixed (suggesting a chromosome evolutionary stasis), while in other genera such as *Calumma*, *Trioceros* is well represented along with karyotypes with a lower, or much more rarely, with a higher chromosome count [10,12].

It should also be noted that the independent nature of the chromosome fusions occurring in and within different chameleon genera generally means that these macromutations should often be considered non-homologous, involving different micro- and macrochromosome pairs [12]. This is often indicated by several cytogenetic characteristics, such as a different micro-/macrochromosome ratio in karyotypes with the same diploid number, a different morphology of macrochromosome pairs, and the variable chromosome location of cytogenetic markers (see [10,11,12]). For instance, the position of NORs on microchromosomes is generally considered a plesiomorphic karyotype state in squamates [25,26], and it is invariably found in *Brookesia* and *Palleon* (see [10,12]). On the contrary, in the genus *Furcifer*, which is characterized by a generally reduced chromosome number (to 2n = 20–22), the NORs are localized on different macrochromosome pairs in different species (often on pair 2, but alternatively on pair 1, 3, 4, and 7), likely as remnants of past micro- to macrochromosome translocations involving distinct pairs (see [9,10,12]).

Overall, the genus *Furcifer* shows some insightful cytogenetic characteristics, which include the highest karyotype variability among chameleons (from 2n = 34 to 2n = 20) and the only known sex chromosome systems with female heterogamety in the whole family (see [9,10,11,12,27]; this study).

**Figure 4:**
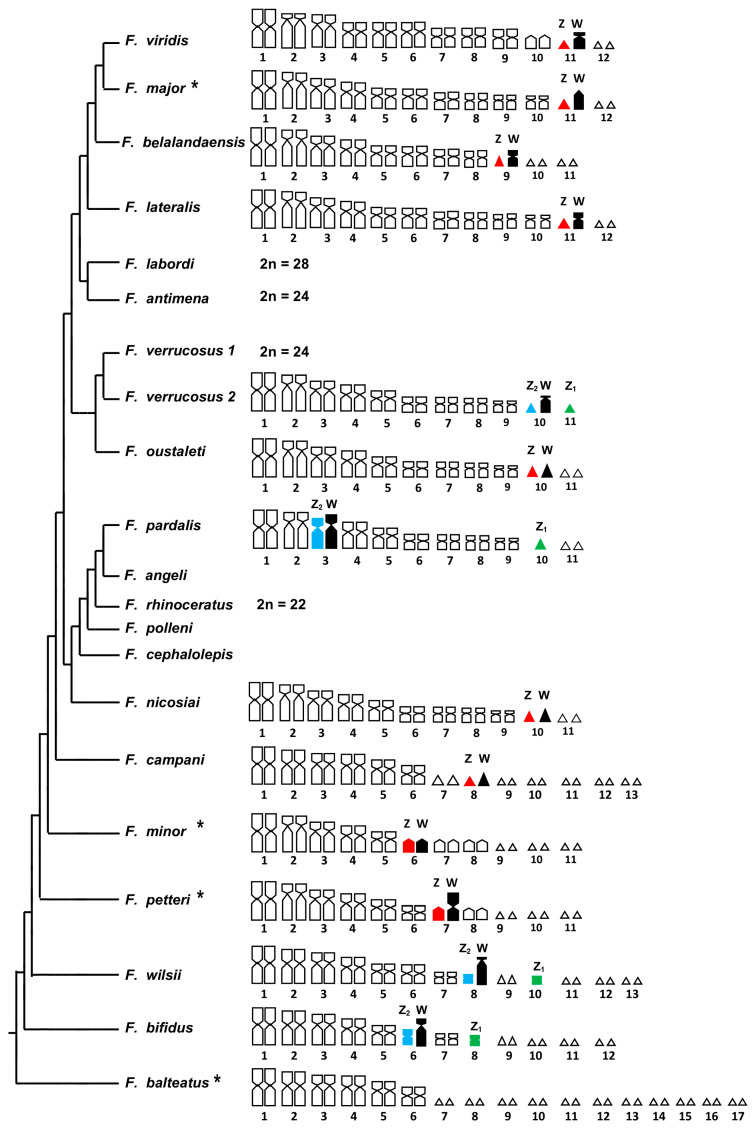
*Furcifer* karyograms with available data on sex chromosome systems superimposed on the phylogenetic relationships of the genus redrawn from Mezzasalma et al. [12] (branch length are for visualization only). Z_1_, Z_2_, and simple Z chromosomes are highlighted in green, blue, and red, respectively. W chromosomes are highlighted in black. Diploid chromosome numbers are shown for species with undetermined sex chromosomes. Cytogenetic data were collected from [9,10,11,12]. Species marked with “*” were analyzed in this study. See text for details.

In particular, excluding *F. balteatus* (see below), all the *Furcifer* species studied with traditional and banding techniques present a single (ZZ/ZW) or multiple (Z_1_Z_1_Z_2_Z_2_/Z_1_Z_2_W) sex chromosome system, which originated from an autosome–sex chromosome (W) fusion (see [9,10,11]; this study) (Figure 4).

Furthermore, recent cytogenomic analysis highlighted that the ZW chromosomes are highly homologous and evolutionary stable in the genus [27].

However, the Z and W chromosomes in *Furcifer* are usually of different shape (in relation to the morphological categories based on the centromeric index proposed by Levan et al. [23]) and/or dimension. In particular, the Z (and Z_1_ and Z_2_) chromosomes are sometimes micro- (e.g., in *F. verrucosus* and *F. oustaleti*) or machrochromosomes (e.g., in *F. bifidus* and *F. minor*) (see [9,11], this study). Furthermore, they range from metacentric (e.g., in *F. bifidus* and *F. wilsii*) to acrocentric (e.g., *F. minor* and *F. lateralis*). Similarly, the W chromosomes alternatively appear as macrochromosomes that are much larger than the Z (e.g., in *F. pardalis*, *F. wilsii*, and *F. verrucosus*) or as microchromosomes that are dimensionally comparable to their pseudohomologs (see [9,11]; this study) (Figure 4).

Interestingly, so far, the only known exception is represented by *F. balteatus,* which has been studied with banding techniques for the first time in this work. This species did not show any differentiated Z/W chromosomes, which are probably still highly homologous and in an early stage of diversification (see Results and Figure 4). Furthermore, *F. balteatus* represents the only known species in the genus with >30 chromosomes and shows a very similar chromosome complement (of 2n = 34 with 6 macro- and 11 micro-pairs) to the ancestral chameleon karyotype of *Brookesia* and *Palleon* (of 2n = 36, see above), from which it probably originated by means of a chromosome fusion of a single microchromosome pair (see also [12]).

Notably, the conservation in *F. balteatus* of a karyotype that is similar to the ancestral 2n = 36 (with the plesiomorphic absence of differentiated sex chromosomes) is also reflected by the phylogenetic position of the species, which is the sister clade to all the other *Furcifer* species (see [12,28]) (Figure 4). It is also nowadays widely accepted that all sex chromosome pairs start their diversification from homomorphic autosomes (the proto sex chromosomes) after the onset of a sex-related mutation in one sex (see, e.g., [29,30,31]. From this starting point, the nucleotide and morphological differences between the ZW (or XY) pairs progressively accumulate by differential heterochromatinization (of the W/Y chromosome) and/or other chromosome mutations (see, e.g., [30,31]).

In particular, from the primitive condition of *F. balteatus*, the evolution of either autosomes and sex chromosomes proceeded in the genus *Furcifer* originating karyotypes with a lower chromosome number (to 2n = 20) and diversified sex chromosome systems via non-homologous chromosome fusions, which could provide a clear explanation of the different size and morphology of the Z chromosomes in distinct species as well as of the independent rise of multiple Z_1_Z_1_Z_2_Z_2_/Z_1_Z_2_W systems in different clades (Figure 4).

As described by the general theories of sex chromosome evolution (see, e.g., [30,31,32]), the diversification of the W elements is likely characterized first by a fast process of heterochromatinization that produces large W chromosomes (e.g., in *F. petteri*, *F. wilsii,* and *F. verrucosus*) and then by a progressive deletion of heterochromatin originating middle-sized elements (e.g., in *F. lateralis*) and the micro-shaped W chromosomes (e.g., in *F. oustaleti* and *F. major*) ([9,10]; this study). Notably, the processes of differential heterochromatinization (amplification/deletion) may happen independently and with different timings in different lineages (e.g., [33]), resulting in different species displaying distinct evolutionary stages. As already described in several squamate taxa (see, e.g., [30,31,32]), W–autosome fusions originated the multiple sex chromosome systems reported in several clades (Figure 4).

It should also be noted that we cannot be sure of the relative dimensions of the Z and W chromosomes in *F. belalandensis*, *F. campani*, *F. nicosiai,* and *F. viridis* because the karyotype of the female individuals studied was not analyzed with banding techniques [12], and therefore the larger element could be alternatively interpreted as either chromosome.

Nevertheless, to the best of our knowledge, *Furcifer* is the only vertebrate genus where all the different hypothesized steps of diversification of heteromorphic sex chromosomes can be observed in different species (including the undifferentiated pair in *F. balteatus*, large heterochromatic and heterogametic chromosomes, heteromorphic sex chromosomes of progressively reduced dimensions, and the independent formation of multiple sex chromosome systems). This evidence provides empiric confirmation on the theory of sex chromosome diversification by heterochromatinization, which has been described several times in different taxa (see, e.g., [30,31,34,35,36]).

The diversification of autosomes and Z/W chromosomes in *Furcifer* via non-homologous fusions could also have promoted speciation by establishing postzygotic reproductive barriers among species with partially homologous neo-formed chromosomes (e.g., [37]). The whole evolutionary scenario described above could be further tested with molecular cytogenetic techniques on a wider taxon sampling that could be useful in reconstructing the distinct autosome–autosome and Z/W–autosome fusions in different clades.

## 5. Conclusions

The genus *Furcifer* represents a particularly interesting case in evolutionary cytogenetics. Different clades of the genus are characterized by a high chromosome diversity in terms of chromosome number (2n = 20–34), morphology, and the formation of differentiated simple (ZZ/ZW) and multiple sex chromosome systems with female heterogamety (Z_1_Z_1_Z_2_Z_2_/Z_1_Z_2_W). Although high molecular homology has been found among sex chromosomes in different *Furcifer* species, either the Z or the W chromosomes are often of different size and morphology. Chromosome evolution in the genus has probably started from a karyotype similar to that of *F. balteatus* (2n = 34 with 6 macro- and 11 microchromosome pairs and no differentiated sex chromosomes), which only slightly differs from the hypothesized ancestral karyotype of the whole family Chamaeleonidae (2n = 36 with 6 macro- and 12 microchromosome pairs). The chromosome evolution of the other species of the genus has been characterized by a progressive reduction of the chromosome number (to 2n = 20) mostly by non-homologous autosome–autosome, Z–autosome, and W–autosome fusions.

## Figures and Tables

**Table 1:** Taxonomic attribution, origin, sex, field number of the individuals studied in this work.

Species	Specimen	Locality	Sex
*B. superciliaris*	GA 199	Fiherenana	female
*B. superciliaris*	GA A3	Fiherenana	male
*F. balteatus*	GA 379	Ranomafana	female
*F. petteri*	FGMV 3015	Ifaty	female
*F. minor*	GA 489	Antoetra	female
*F. major*	GA 456	Belalanda	female

## Data Availability

The newly generated DNA sequences were deposited to GenBank (accession numbers: PQ272538-43).
